# Food expenditure patterns in the Canadian Arctic show cause for concern for obesity and chronic disease

**DOI:** 10.1186/1479-5868-11-51

**Published:** 2014-04-17

**Authors:** Mohammadreza Pakseresht, Rosalyn Lang, Stacey Rittmueller, Cindy Roache, Tony Sheehy, Malek Batal, Andre Corriveau, Sangita Sharma

**Affiliations:** 1 Aboriginal and Global Health Research Group, Department of Medicine, University of Alberta, 5–10 University Terrace, Edmonton, AB T6G 2 T4, Canada; 2 North Carolina A&T State University, Department of Biology, African Americans & Alzheimer's Disease Research Study, 2105 Yanceyville Building, Greensboro, NC, 27410, USA; 3 School of Food and Nutritional Sciences, University College Cork, Cork, Ireland; 4 Département de nutrition, Faculté de medicine, Université de Montréal, C.P. 6128, Succursale Centre-ville, Montréal, Québec, H3C 3 J7, Canada; 5 The Northwest Territories Department of Health and Social Services, Yellowknife, NT, X1A 2 L9, Canada; 6 Present address: Aboriginal and Global Health Research Group, Department of Medicine, University of Alberta, 5-10 University Terrace, Edmonton, AB, T6G 2 T4, Canada

## Abstract

**Background:**

Little is understood about the economic factors that have influenced the nutrition transition from traditional to store-bought foods that are typically high in fat and sugar amongst people living in the Canadian Arctic. This study aims to determine the pattern of household food expenditure in the Canadian Arctic.

**Method:**

Local food prices were collected over 12 months in six communities in Nunavut and the Northwest Territories. Dietary intake data were collected from 441 adults using a validated quantitative food frequency questionnaire. Money spent on six food groups was calculated along with the cost of energy and selected nutrients per person.

**Results:**

Participants spent approximately 10% of total food expenditure on each of the food groups of fruit/vegetables, grains and potatoes, and dairy, 17% on traditional meats (e.g. caribou, goose, char, and seal liver), and 20% on non-traditional meats (e.g. beef, pork, chicken, fish, and processed meats). Non-nutrient-dense foods (NNDF) accounted for 34% of food expenditure. Younger participants (<30 years) spent more on NNDF and less on traditional meats compared with the older age groups. Participants with higher levels of formal education spent more on fruit and vegetables and less on traditional meats, when compared with participants with lower levels of formal education.

**Conclusions:**

Participants spent most household income on NNDF, a possible consequence of generation discrepancy between younger and older participants. The tendency toward NNDF, particularly among youth, should be addressed with an assessment of predictive factors and the development of targeted approaches to population-based interventions.

## Introduction

Between 2000 and 2004, the average mortality rates due to cardiovascular disease (CVD) in Nunavut (NU) and the Northwest Territories (NWT) were higher than the national average [1]. A cancer statistics report for the period of 2003 and 2007 shows that the annual cancer incidence rate was higher in these two territories compared to the national rate [2]. For a period of 10 years (1991–2000) the age-standardized incidence rate of cancer increased by 11% in the NWT and 29% in NU [3]. The crude prevalence rates of diagnosed diabetes among the population ≥ 20 years in NU and the NWT were lower than the Canadian average [1]. This lower rate could be partially related to an under diagnosis of patients with diabetes. However, the prevalence of diabetes in the NWT increased from 1.8% to 4.6% between 1994 and 2010. This rate increased from 1.9% to 3.3% in NU between 2000 and 2010 [3]. In 2011, the age-standardized percentage of obesity, an intermediate risk factor for chronic disease, in the NWT and NU was higher than the national average (26 and 28 vs. 18, respectively) [4].

Aboriginal populations in the Canadian Arctic are experiencing an on-going nutrition transition characterized by decreased traditional food intake and increased reliance on store-bought foods, high in fat, sugar, and energy, which are known for their association with developing chronic diseases and obesity [5]. In the remote and isolated communities of the Canadian Arctic, the cost of purchasing nutritious foods for a household can pose a significant barrier to achieving a healthier diet. Studies have shown that when cost is the main factor in food selection, diets tend to be nutrient-poor and energy-rich [6]. Cost of all essential household goods, including non-food items, should be considered in the context of current dietary and household patterns in order to design an effective, relevant, and culturally appropriate comprehensive program.

Analysis of the food expenditure patterns can provide insight into possible causes of obesity, as a common risk factor for chronic disease. Health providers and policy makers will benefit from this information to provide health advice at individual and population levels. The relationship between food expenditure and diet quality is potentially driving the magnitude and intensity of the nutrition transition in the Canadian Arctic. A greater understanding of the relationship between household food expenditure patterns and dietary habits is required to inform the development of effective nutrition interventions.

The aim of the present study was to determine patterns of expenditure on food groups amongst Inuit and Inuvialuit in the Canadian Arctic.

## Subjects and method

### Setting

The study is based on data collected for the Healthy Foods North project [7] from six communities across two territories in the Canadian Arctic, NU and the NWT. Communities selected for participation represented varying percentages of Inuit or Inuvialuit populations in relation to overall community population and socioeconomic status. The community populations have been previously described [7]. In brief, the NU communities range in population from 800–1,500 people, 80-90% of whom self-identify as Inuit. The median Inuit age ranges from 20–26 years, employment rate ranges from 40-60%, and the median household income is CAD$34,000-60,000 [8]. The three communities in the NWT range from 400–3,500 people with Inuvialuit populations ranging from 40-90%. Median age of Inuvialuit in these communities ranges from 24–26 years, employment rate is 40-65%, and median household income is CAD$33,000-64,000 [8]. Each of the six communities has two to three food stores that obtain food primarily through shipments from the south via airplane year round, via roads and/or ice roads for part of the year (NWT only), and via barge or sea lift once per year when the sea ice melts and shipping routes by sea are open. Food is also obtained, to varying degrees, by traditional means (e.g. hunting, fishing, food sharing networks).

### Data collection and analysis

Adult participants (≥19 years) who resided in the community for more than six months, excluding pregnant/lactating women due to their different nutritional requirements and dietary habits, were recruited by random selection using the up-to-date community housing maps provided. If nobody was available in the randomly selected house after three attempts through in person contact, the next house was chosen. A random house was substituted if the eligible subject from the initially selected house declined to participate. This method ensured sampling from areas with varied proximities to food stores. One resident per household, ideally the person who was the main food shopper/preparer, was recruited. Exclusion criteria included pregnant/lactating women, due to this group’s different nutritional requirements and possible changes in dietary habits. Response rates in NU communities ranged from 69-93% and 65-85% in NWT communities.

A culturally appropriate quantitative food frequency questionnaire (QFFQ) was developed [9,10] and validated [11,12] for each territory separately. The two FFQs had the same structure to record the frequency of intake, portion size, and number of portions consumed in each setting, but each included a different number of food items (150 and 142 food items in the NU and NWT FFQs, respectively). The questionnaires were administered to 211 Inuit in NU and 230 Inuvialuit in the NWT. Information from Canadian food composition tables [13], locally collected recipes and the USDA National Nutrient Database for Standard Reference [14] were used to construct a food composition table specifically for each QFFQ. A record that contained energy and nutrient content per 100 grams for each food item in the QFFQ was created in the relevant food composition table. The data extracted from three datasets, including the food composition table, QFFQ and food item portion weights, were analysed.

Project staff were trained by the principal investigator (S.S.) to record grocery store prices for each food item listed in the QFFQ. Food prices were collected approximately once a month between June 2008 and November 2009 from at least two food stores in each community. The prices of one to five brands of each food item were recorded, based on store availability, to capture a range of prices. The average prices of different brands were considered for further analysis. The weight (grams) and price in CAD$ of all food items were recorded per package. Data on costs for traditional meats were obtained from local hunters through the Hunters and Trappers Organization and accounted for the cost of equipment and supplies. All food items in the QFFQ were categorized into six main food groups [fruit and vegetables, grains and potatoes, dairy, traditional meats, non-traditional meats, and non-nutrient-dense foods (NNDF)] based on a strategy developed for previous publications for this study [15,16] (Table 1). Food items in the NNDF category included all foods that did not fall into other food groups and supplied less than 5% of the reference daily intakes per serving for protein, calcium, iron, and vitamins A, C, B_1_, B_2_, and niacin [17].

**Table 1:** Food groups, subgroups and number and example of food items in each group

**Food groups**	**Food items (n)**	**Food subgroups (food items example)**
Dairy	11	Milks, cheeses, yogurts, eggs
Grains & potatoes	16	White breads, whole wheat, cereals, noodles, rice, potatoes, crisps
Fruit & vegetables	21	Fresh fruits, packaged fruits, vegetables
Non-traditional meats	26	Beef/pork, chicken/turkey, canned fish, soups/stews
Traditional meats (only meats and animal organs)	39	Land (caribou, muskox, polar bear), sea (seal, muktuk, char), sky (goose, ptarmigan)
Non-nutrient-dense foods	26	High fat/high sugar foods (butter, pizza, popcorn, juice sweetened, ice cream, chocolates)

A supplementary questionnaire was used to collect information regarding sex, age, weight, height, smoking status, level of education, and the Material Style of Life (MSL) scale. As a proxy for socioeconomic status, the MSL scale assessed whether a participant’s household owned a series of 20 items of varying costs in working condition (e.g. television, snowmobile) [18].

Institutional Review Board approval was obtained from the Committee on Human Studies at the University of Hawaii and the Office of Human Research Ethics at the University of North Carolina at Chapel Hill. The Ethics Committee of the Beaufort Delta Health and Social Services Authority also approved this project and the Aurora Research Institute in the NWT and the Nunavut Research Institute in NU provided research licences.

### Data analysis

Energy cost for each food group was described as the total cost spent on food items belonging to a food group to obtain optimal energy intake. Average daily consumption of each food item and the amount spent per food item per day was computed for each participant. For each FFQ item, total cost per 100 grams of each food item was calculated by dividing the average of the item’s price over a 12 month period by the final unit weight (grams) and multiplying by 100.

Only formal levels of education were recorded during data collection and the valuable traditional culture knowledge and education was not considered in the study. Level of education was categorized into low (none or some elementary school, completed elementary school or some junior high school), intermediate (completed junior high school, some high school or completed high school), and high (some college, trade school or some university or university completed). The MSL scale was also categorized into low (0–7), intermediate (8-12), and high (13-20).

Data were analyzed using STATA, version 11 (StataCorp LP, College Station, Texas, USA). Student *t*-test and chi-squared test or one-way ANOVA were used to examine null hypotheses. To reduce the chances of obtaining false-positive results (type I errors) by performing multiple pair wise tests on the study data, all *P*-values were considered statistically significant at α < 0.01.

## Findings

The study sample included 441 participants (80% of whom were women). One hundred and twenty six (29%) participants were ≥50 years and 77 (17%) participants were under 30 years of age; the mean age was 43 (SD 14) years. Twenty three percent of participants were overweight, 47% were obese, and 71% were smokers. Overall, participants spent an average of CAD$7,217 per year (CAD$19.7 per day) on food. Men, participants under 30 years, participants with an intermediate level of education, and those who smoked spent significantly more on foods compared with other subgroups (Table 2).

**Table 2:** **Demographic information and annual expenditure on foods**^†^**for Inuit and Inuvialuit populations**

	**n (%)**	**CAD$/yr**	***P value***
		**Mean (95% CI)**	
**All participants**	441 (100)	7217 (6845, 7586)	
**Sex**			
Men	87 (20)	8483 (7558, 9407)	<0.001^¥^
Women	354 (80)	6904 (6506, 7301)	
**Age groups**			
<30 y	77 (17)	8907 (7880, 9934)	<0.001^§^
30-49 y	238 (54)	7666 (7178, 8155)	
≥50 y	126 (29)	5329 (4792, 5866)	
**Education**			
Low	159 (37)	6275 (5771, 6779)	<0.001^§^
Intermediate	184 (42)	7877 (7257, 8497)	
High	89 (21)	7517 (6619, 8414)	
**BMI** (kg/m^2^)			
< 25	118 (30)	7288 (6603, 7960)	0.12^§^
25 - <30	90 (23)	7846 (6923, 8769)	
≥ 30	189 (47)	6834 (6300, 7368)	
**Smoking**			
No	127 (29)	5769 (5171, 6367)	<0.001^¥^
Yes	314 (71)	7766 (7315, 8217)	
**MSL score**^‡^			
Low	127 (29)	7306 (6587, 8024)	0.42^§^
Intermediate	151 (35)	6875 (6317, 7433)	
High	155 (36)	7455 (6772, 8137)	

^†^Overall food expenditure for six main food groups: fruit & vegetables, grains & potatoes, dairy, traditional meats, non-traditional meats, and non-nutrient-dense foods.

^‡^Low: 0–7, Intermediate: 8–12, High: 13–20.

^¥^*P* value for Student *t*-test for food expenditure.

^§^*P* value for One way ANOVA for food expenditure.

MSL: Material Style of Life.

**Figure 1:**
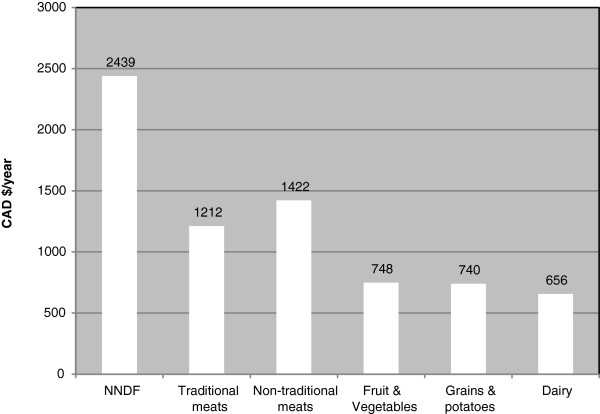
**Average annual food expenditure (CAD$) by Inuit and Inuvialuit for six food groups.** NNDF: non-nutrient-dense foods.

Participants spent the most money on NNDF (CAD$2,439) followed by non-traditional meats (CAD$1,422) and traditional meats (CAD$1,212) (33.8%, 19.7%, and 16.8% of food expenditure, respectively) (Figure 1 & Table 3). Less than CAD$800 per year was spent on fruit and vegetables, grains and potatoes, or dairy (10.3%, 10.3%, and 9.1% of food expenditure, respectively). Men, compared with women, spent more on non-traditional meats (24.8% vs. 18.5% of their food expenditure, p< 0.001). Participants ≥50 spent more on traditional meats compared with the younger groups (21.3% vs. 10.8% and 16.3%, p < 0.001), but younger individuals tended to pay more for NNDF (41.0% vs. 33.9% and 29.2%, p < 0.001). Participants who were categorized with a higher level of formal education spent more on fruit and vegetables (12.8% vs. 9.4% and 9.6%, p = 0.004) but less on traditional meats (13.7% vs. 21.3% and 14.2%, p < 0.001) compared with other participants.

**Table 3:** Proportion of food expenditure for food groups based on demographic variable categories

	**Fruit & vegetables % (95% CI)**	**Grains & potatoes % (95% CI)**	**Dairy % (95% CI)**	**Non-traditional meats % (95% CI)**	**Traditional meats % (95% CI)**	**NNDF % (95% CI)**
**All participants**	10.3 (9.5, 11.0)	10.3 (9.9, 10.8)	9.1 (8.3, 9.8)	19.7 (18.7, 20.8)	16. 8 (15.5, 18.1)	33.8 (32.3, 35.3)
**Sex**						
Men	8 (6.8, 10.4)	9 (8.9, 10.8)	8 (6.8, 9.4)	24 (22.2, 27.4)	13.6 (10.9, 16.3)	35.0 (31.6, 38.4)
Women	10.6 (9.8, 11.5)	10.5 (10.0, 11.0)	9.3 (8.4, 10.2)	18.5 (17.4, 19.6)	17.5 (16.1, 19.0)	33.5 (31.8, 35.2)
*P* value^†^	0. 03	0 . 27	0 . 21	<0 . 001	0 . 02	0 . 44
**Age groups**						
<30 y	8.2 (6.4, 10.0)	9.4 (8.3, 10.5)	10.7 (8.4, 12.9)	20.0 (17.3, 22.6)	10.8 (8.2, 13.4)	41.0 (36.8, 45.1)
30-49 y	10.5 (9.4, 11.5)	10.4 (9.7, 11.0)	8.5 (7.6, 9.4)	20.4 (19.0, 21.8)	16.3 (14.7, 17.9)	33.9 (32.0, 35.8)
≥50 y	11.0 (9.6, 12.5)	11.0 (10.2, 11.9)	9.2 (7.7, 10.7)	18.3 (16.4, 20.2)	21.3 (18.5, 24.0)	29.2 (26.4, 31.9)
*P* value^‡^	0.04	0.08	0.11	0.23	<0.001	<0.001
**Education**						
Low	9.4 (8.4, 10.5)	10.7 (9.9, 11.6)	8.1 (7.0, 9.2)	18.7 (17.0, 20.5)	21.3 (19.0, 23.6)	31.7 (29.2, 34.1)
Intermediate	9.6 (8.6, 10.7)	10.2 (9.5, 10.9)	9.9 (8.6, 11.2)	19.7 (18.1, 21.2)	14.2 (12.3, 16.0)	36.3 (33.9, 38.8)
High	12.8 (10.5, 15.1)	9.8 (8.9, 10.7)	8.8 (7.2, 10.4)	21.5 (18.9, 24.1)	13.7 (11.2, 16.2)	33.5 (30.2, 36.7)
*P* value^‡^	0.004	0.33	0.11	0.18	<0.001	0.03
**BMI** (kg/m^2^)						
< 25	9.0 (7.9, 10.2)	115 (10.5, 12.6)	10.0 (8.2, 11.9)	18.6 (16.7, 20.4)	15.9 (13.4, 18.3)	34.9 (32.0, 37.9)
25 - <30	9.5 (8.1, 11.0)	9.9 (9.0, 10.9)	8.2 (6.9, 9.6)	20.4 (18.0, 22.8)	16.1 (13.3, 18.9)	35.8 (32.4, 39.2)
≥ 30	11.9 (10.5, 13.3)	10.1 (9.5, 10.7)	8.7 (7.7, 9.8)	19.7 (18.1, 21.4)	17.9 (15.9, 19.9)	31.6 (29.3, 33.9)
*P* value^‡^	0.01	0.02	0.23	0.48	0.37	0.07
**Smoking**						
No	11.0 (9.7, 12.3)	10.7 (9.9, 11.6)	8.6 (7.2, 9.9)	18.5 (16.7, 20.2)	18.8 (16.3, 21.3)	32. (29.6, 35.2)
Yes	10.0 (9.0, 10.9)	10.2 (9.6, 10.8)	9.3 (8.4, 10.2)	20.3 (19.0, 21.6)	15.8 (14.3, 17.3)	34.4 (32.6, 36.2)
*P* value^†^	0.25	0.29	0.40	0.11	0.04	0.25
**MSL score**^¥^						
Low	8.6 (7.4, 9.8)	10.6 (9.6, 11.6)	10.2 (8.5, 11.9)	20.1 (18.0, 22.2)	15.0 (12.8, 17.2)	35.4 (32.3, 38.6)
Intermediate	11.0 (9.5, 12.6)	10.3 (9.5, 11.1)	9.0 (7.7, 10.3)	20.9 (19.1, 22.8)	16.0 (13.6, 18.3)	32.7 (30.3, 35.2)
High	10.7 (9.5, 11.9)	10.1 (9.4, 10.9)	8.0 (7.0, 9.1)	18.3 (16.7, 19.8)	18.9 (16.7, 21.0)	34.0 (31.5, 36.5)
*P* value^‡^	0.03	0.75	0.08	0.10	0.04	0.38

^†^*P* value for Student *t*-test.

^‡^*P* value for One way ANOVA.

^¥^Low: 0–7, Intermediate: 8–12, High: 13–20.

MSL: Material Style of Life.

NNDF: non-nutrient-dense foods.

No statistically significant difference was observed for food group expenditure amongst participants in various BMI, MSL score, and smoking categories.

## Interpretation

To the best of our knowledge, this is the first study to examine food expenditure patterns in two territories in the Canadian Arctic (NU and the NWT). About 34% of total food expenditure by participants was spent on NNDF. Participants also spent more money on non-traditional meats and traditional meats, compared with fruit and vegetables, grains and potatoes, and dairy products.

The findings of the present study raise significant questions concerning the disproportionate amount of money being spent on NNDF, which do not provide adequate nutrition, but provide relatively high levels of energy. NNDF includes a relatively large group of items that were usually available in community food stores, in contrast to fresh produce, which was not as readily available on a consistent basis. This suggests that, on a daily basis, most of the NNDF items were more available than fresh produce. For individuals who are food shopping for the entire family, the purchase of NNDF may be the easiest way of obtaining the most energy while ensuring that all members of the family were satisfied. In addition, a taste preference may exist for high fat foods [19]. Drewnowski and Specter [20] reported that the relationship between obesity and poverty can be explained by the low cost of foods that are energy dense and the taste and acceptability of these foods that are usually high in fat and sugar. Affordability, quality of store-bought perishable foods, exposure to fresh produce, and culinary knowledge are other factors that could be linked to lower expenditure on nutrient-dense food groups, particularly fruit and vegetables. Increased cost of fuel and hunting equipment, as well as reduced availability of land, sea and sky resources, such as caribou, Arctic char, and geese, possibly as a result of climate changes [21], are potential reasons that traditional meats expenditure is lower than non-traditional meat expenditure among this population.

Food group expenditure in this study varied among age, gender, and education subgroups. This finding is supported by results from an Expenditure Survey conducted by the U.S. Bureau of Labor Statistics [22]. The reality in traditional and remote communities is that men generally spend more time outside the household for work and have less food-preparation skills than women; this could explain the gender difference of non-traditional meat food expenditure in this study. Physiological need difference between genders for protein and energy expenditure could also partly explain the variety of meat intake and expenditure between men and women in the study population [23]. The finding that women consume diets with less meat reported by other studies [24,25] support our result.

Our analysis showed a statistically significant, increasing trend for traditional meats expenditure and a decreasing trend for NNDF expenditure with an increase of age. Similarly, in the study by Fan *et al.*[22] older households were less likely to be in the fast-food-dominant food expenditure category. Fast food tends to be more energy dense, higher in saturated fat and salt, and lower in micronutrients relative to other foods [26] and thus, could be considered equivalent to NNDF in this study. It is known that dietary habits tend to be relatively stable over time [27], suggesting that older people may have a higher tendency towards traditional meats in these communities.

The finding that participants with higher levels of formal education spent significantly more on fruit and vegetables is comparable with the results from the East Anglia cohort of the European Prospective Investigation of Cancer analysis [27]. This finding could be linked to having greater nutrition knowledge regarding fruit and vegetables, being more familiar with method of preparing dishes using fruit and vegetables, and more taste preference for this food group*.* A high level of expenditure on traditional meats by people classified with a lower level of formal education is compatible with the similar expenditure pattern and amount among older participants (both groups spent 21% of the food expenditure on traditional meats). This indicates a preference towards traditional meats is likely related to interest in and commitment to customs as well as an awareness of nutritional values of traditional meats.

In 2007–2008, approximately 7.7% of Canadian households and 21% of off-reserve Aboriginal households were food insecure [28]. In one recent survey, nearly 70% of Inuit families in 16 NU communities were food insecure [29]. Previous studies among this population indicate that this may be an issue among Inuit and Inuvialuit [7]. In other populations, individuals who are food insecure and have a low income often rely heavily on low-cost foods that are high in energy (sugar and fat) but lack essential nutrients [20,30]. In this study, however, no difference in patterns of food expenditure was found between different levels of socioeconomic status (MSL scale), suggesting factors such as taste preference or availability may have more of an influence on food expenditure patterns, at least among people with adequate income. In addition, this finding indicates that any future intervention program could be broadly applied to all members of the community.

Nutrition related diseases such as obesity and diet related anemia can be caused by an insufficient intake of food, insufficient intake of certain nutrients or by an over consumption of certain foods [31]. Addressing dietary inadequacies through appropriate and relevant intervention programming has the potential to positively impact health status and to reduce the cost to health systems in the Canadian Arctic.

Municipal, regional, territorial, and federal governments and organizations should consider the findings of this study in the development, design, and implementation of any programming or subsidies related to the food environment in the Canadian Arctic. Particular focus should be given to costs associated with hunting and gathering and supportive programs for younger generations of Inuit/Inuvialuit to learn hunting and gathering skills.

All future programming should consider challenges to obtaining a healthy diet in the Canadian Arctic, whether they relate to store-bought or traditional meats. High costs associated with household essentials, such as diapers and toilet paper, can take away from the disposable household income available for the purchase of food. As such, given the importance of traditional meats to the diet of Inuit/Inuvialuit, subsidies should be provided to reduce costs associated with hunting, such as snowmobile parts, ammunition, and fishing supplies. Any programs or subsidies should include rigorous community consultation to ensure programs are relevant and appropriate for the current Northern environment. Communities that are to receive the program and/or subsidy should endorse all promoted items.

Considering the findings of this study in relation to food choices and costs, programs should be developed to promote food items that have the largest impact on diet and be the most amenable to Inuit/Inuvialuit communities, such as eggs and yogurt. In addition, the current study shows that younger people tended to pay more for NNDF. Thus, particular emphasis should be paid to engaging young people in the development and implementation of household and individual budgeting support, in addition to healthy food choices, as a key element of any intervention program.

Further research into all factors impacting overall household income in the Canadian Arctic is required to ensure any program and policy decisions are appropriate, relevant, and timely. If a large percentage of household income is spent on NNDFs, then the available disposable income will decrease, considering a subsidy of only healthy food choices. Without community-led programs that support healthy food choices, simply subsidizing the cost of certain foods will continue to increase the likelihood of food insecurity in the Canadian Arctic.

Food prices were sampled over a 12-month period and therefore, variations in food costs due to seasonality and for different brands were captured. In addition, there was very limited diversity for food item brands between communities due to remoteness. Women contributed more than men in the study because the study preference was to recruit a member of each household who was the main person shopping for and preparing food for the family. This might limit the ability to generalize the findings to people living outside of these communities. However, it was necessary to collect detailed and accurate dietary information from participants to address the HFN project’s objectives. This need could be addressed by recruiting main food shoppers from each household as those people were more likely to have food knowledge and manage food expenditure for the household. Accordingly, collecting more accurate dietary data was more advantageous to the study compared with the disadvantage associated with potential biases. Majority (54%) of participants were between 30 and 49 years. This matches with the age pyramid in these communities where 46% of the population in the six communities was also between 30 and 49 years. A chi-squared test also did not show any significant difference between communities and the study participants for the proportion of people in the three age groups (*P* = 0.19). However, women usually attach greater importance to healthy eating (e.g. more fibre and less fat intake) [32]. Only formal levels of education were recorded during data collection because the valuable traditional culture knowledge and education was not easily measurable.

## Conclusion

High expenditure on NNDF is the main finding of this study. Predictive factors of high expenditure on NNDF and opportunities for reduced spending on NNDF and barriers for increased spending on traditional meats should be understood before any intervention to improve dietary situation in this population. Educational efforts regarding healthy eating choices should focus on younger age groups. In addition, improvement in household income, reduction in cost of hunting and fishing, and availability of traditional meats (i.e. declining herds) would support any healthy eating intervention program in Canadian Arctic.

## Competing interests

The authors declare that they have no competing interests.

## Authors’ contributions

MP did data analysis, data interpretation, literature search, and wrote the article. RL and SR collected the data, reviewed literature and drafted the initial version of the article. CR, TS, MB and AC contributed in data interpretation and writing the article. SS designed the study and was principal investigator of the project. She supervised all stages of the project and writing this article. All authors read and approved the final manuscript.
